# Newly Identified TPI Deficiency Treatments Function for Novel Disease-Causing Allele, *TPI1^R5G^*

**DOI:** 10.3390/genes16101205

**Published:** 2025-10-14

**Authors:** Joseph R. Figura, Presley Roberts, Riley Sawka, Maci Chambers, Marcelo Claudio, Laura L. Vollmer, Andreas Vogt, Gregg E. Homanics, Eduard van Beers, Mylene Donge, Emmanuel Scalais, Arthur Sorlin, Ariana J. Jou, Andrew P. VanDemark, Michael J. Palladino

**Affiliations:** 1 Department of Pharmacology & Chemical Biology, University of Pittsburgh School of Medicine, Pittsburgh, PA 15261, USA; jof149@pitt.edu (J.R.F.); pmr45@pitt.edu (P.R.); ris227@pitt.edu (R.S.); mjc221@pitt.edu (M.C.); marcelo.claudio@upr.edu (M.C.); 2 Pittsburgh Institute for Neurodegenerative Diseases, University of Pittsburgh School of Medicine, Pittsburgh, PA 15260, USA; 3 Department of Computational & Systems Biology, Organ Pathobiology and Therapeutics Institute, University of Pittsburgh School of Medicine, Pittsburgh, PA 15261, USA; llv4@pitt.edu (L.L.V.); avogt@pitt.edu (A.V.); 4 Department of Anesthesiology and Perioperative Medicine, University of Pittsburgh, Pittsburgh, PA 15261, USA; geh2@pitt.edu; 5 Department of Haematology, Center for Benign Haematology, Thrombosis and Haemostasis, University Medical Centre, 3511 ZK Utrecht, The Netherlands; 6 Pediatric Neurology Unit, Department of Pediatrics, Centre Hospitalier de Luxembourg, L-1210 Luxembourg, Luxembourgscalais.emmanuel@chl.lu (E.S.); 7 Service de Génétique Clinique, National Center of Genetics (NCG), Laboratoire National de Santé, L-3555 Dudelange, Luxembourg; arthur.sorlin@lns.etat.lu; 8 Department of Biological Sciences, University of Pittsburgh, Pittsburgh, PA 15260, USA; ajj58@pitt.edu (A.J.J.); andyv@pitt.edu (A.P.V.)

**Keywords:** triosephosphate isomerase (TPI), pediatric disease, glycolytic dysfunction, biochemistry, metabolism, genetics, small molecule therapy

## Abstract

**Background/Objectives:** Triosephosphate Isomerase (TPI) is a glycolytic enzyme known to be associated with TPI deficiency, a severe form of childhood-onset glycolytic enzymopathy associated with hemolytic anemia, neuromuscular impairment and early death. Most often the disease results from the common *TPI1^E105D^* mutation, which can be either homozygous or compound heterozygous with another allele. **Methods:** We purified *TPI^R5G^* protein, studied mutant protein biochemistry, established and characterized *TPI^R5G/f.s.^*patient cells, and investigated newly identified compounds for their efficacy in vitro using Western blot and TPI activity assays. **Results:** We identified novel *TPI1* alleles that result in TPI Deficiency with an atypical presentation lacking anemia and with more slowly developing neurologic and locomotor impairment. The patient was found to be compound heterozygous with a missense mutation resulting in an R5G amino acid substitution and a frameshift mutation that is a predicted null allele. To better understand disease pathogenesis in this patient, we expressed and purified the TPI^R5G^ human protein and studied it biochemically in addition to studying *TPI^R5G/f.s.^*patient cells. We discovered that purified TPI^R5G^ protein has wildtype activity with modestly increased dimer stability. We also discovered that steady-state TPI protein levels were markedly reduced, suggesting that the instability of the mutant protein underlies disease pathogenesis. We tested compounds recently identified in a screen for novel TPI Df therapies for their efficacy in *TPI^R5G/f.s.^*patient cells. All three compounds significantly increased TPI protein levels in patient cells. As expected, since the mutant protein retains essentially wild type activity, the increase in TPI protein levels also resulted in a significant increase in TPI activity. **Conclusions:** These results establish *TPI^R5G^* as a TPI Df allele, demonstrate that reduced stability of the mutant protein underlies pathogenesis akin to other disease-causing alleles, and suggest that recently discovered developing therapies will likely function broadly and should be developed as potential TPI Df therapies.

## 1. Introduction

Triosephosphate isomerase deficiency (TPI Df) is a rare metabolic disorder characterized by severe neuromuscular dysfunction and early lethality [1,2]. Severe forms of the disease, such as the common mutation with a null allele, result in neonatal-onset disease and lethality in infancy [3]; however, more typically, patients are normal at birth and TPI Df presents early in childhood with the first symptoms being hemolytic anemia and increased rate of infection. As the disease advances, patients experience progressive muscle degeneration and neuronal dysfunction. Weakness and atrophy of both core and limb muscles lead to mobility issues and, in many cases, compromised respiratory function due to diaphragm involvement. Early-childhood death is common as both neurologic and muscular dysfunction progress quickly.

TPI is an essential enzyme involved in both glycolysis and gluconeogenesis, catalyzing the interconversion of dihydroxyacetone phosphate (DHAP) and glyceraldehyde-3-phosphate (G3P). The conversion of DHAP to G3P is crucial for maximizing energy production during glycolysis, as only G3P can be metabolized by the ATP-generating steps in glycolysis. Known to be a perfect enzyme, TPI’s catalytic rate is only limited by the diffusion rate of substrate to its active site [4]. Although overreliance on non-glycolytic sources of ATP production may contribute to disease pathogenesis, there is considerable evidence that DHAP accumulation also contributes, likely through the elevation of methylglyoxal (MGO) [5,6]. MGO is a highly reactive di-carbonyl that likely causes non-specific proteolytic toxicity, but it has also been observed that MGO induces non-enzymatic post-translational modifications in TPI that further reduce the mutant protein’s stability [5]. Although the extent to which this contributes to disease pathogenesis is not fully understood, it would create a pathogenic positive feedback cycle where the mutant protein has reduced stability and is deficient (reduced cellular levels), which leads to increases in DHAP and MGO, resulting in post-translational modifications to TPI that further reduce the stability and levels of the protein. Such a process could contribute to the rapidly progressing nature of this disease.

TPI Df is an autosomal recessive disorder caused by missense mutations in the *TPI1* gene, located on the short arm of chromosome 12, at the 12p13 location. There is only one gene known to encode TPI in humans, which produces three predicted isoforms varying only at the N-terminus with only the canonical ~25 kDa isoform being observed experimentally. A single amino acid substitution at the 105th codon from glutamate to aspartate (*TPI^E105D^*) is found in ~80% of TPI Df patients and is known as the “common” mutation [7]. However, other disease-causing missense mutations, such as *TPI^Q181P^* and *TPI^R190Q^*, have been identified and studied and are typically found as compound heterozygotic mutations with *TPI^E105D^* [8,9].

Currently, there are no pharmacological treatments available for TPI Df. Symptomatic treatment is limited to dietary and nutritional interventions with uncertain therapeutic benefit. Pathogenic disease-causing missense alleles of *TPI1* studied to date all preserve significant catalytic function and cause disease by destabilizing the protein, leading to reduced steady-state levels, presumably due to accelerated degradation, as has been reported for the *TPI^sugarkill^* allele [10,11,12]. Thus, increasing mutant protein levels would be a viable therapeutic approach. To enable screening for potential small molecule therapeutics, human cells stably expressing TPI^E105D^ fused to an eGFP have been generated [13]. Additionally, this methodology was refined into a High Throughput Screen (HTS) that identified several novel classes of lead compounds as potential TPI Df treatments [14].

In this manuscript we report the discovery of a new TPI Df missense allele, *TPI^R5G^*, that causes TPI Df as a compound heterozygote with a frameshift allele (*TPI^f.s.^*). The *TPI^R5G/f.s.^*allelic combination results in TPI Df with locomotor and neurologic symptoms but lacking anemia. As with other pathogenic mutant TPI proteins resulting from missense mutations, the TPI^R5G^ retains significant and essentially wildtype levels of protein activity, and the disease results from the protein’s low steady-state levels, presumably owing to accelerated turnover of the mutant protein. Recently identified developing TPI Df treatments that significantly increase TPI^E105D^ were found to also increase TPI^R5G^ protein levels and TPI activity in patient fibroblasts. These data support the conclusion that *TPI^R5G^* is a novel TPI Df allele and, as in the case of the common *TPI^E105D^*allele, small molecules that increase mutant protein levels should be developed as they could significantly mitigate disease severity in patients with this allele.

## 2. Materials and Methods

### 2.1. Cloning, Expression, and Purification of Recombinant TPIs

The coding sequences of *TPI* wildtype and R5G alleles were cloned into pLC3 vectors encoding the expression of TPI containing a His6-MBP tag that is cleavable by TEV protease. Protein expression was performed in BL21 (DE3) Codon + RIPL *Escherichia coli*, at room temperature through the addition of 0.2 mM isopropyl β-D-1-thiogalactopyranoside (IPTG). After harvesting, the cells were lysed by homogenization in 25 mM Tris-HCl pH 8, 500 mM NaCl, 10% glycerol, 5 mM imidazole, and 1 mM 2-mercaptoethanol (β-ME). Cellular debris was removed by centrifugation at 30,000× *g* for 30 min. TPI protein was purified using nickel-affinity chromatography, followed by digestion with TEV protease to liberate the His_6_-MBP tag from TPI. A second round of nickel-affinity chromatography was performed, followed by anion-exchange chromatography and size-exclusion chromatography (SEC) for further purification. The final protein fractions were concentrated to 6–8 mg/mL and stored at 4 °C for later biochemical assays.

### 2.2. Oligomeric State Determination

400 µL of the indicated TPI protein was dialyzed into 20 mM Tris-HCl pH 8.8, 200 mM NaCl, and 1 mM β-ME and run on a Superdex-200 HR 10/30 (Cytiva, Marlborough, MA, USA) at 0.4 mL/min flowrate. The absorbance at 280 nm was plotted against retention volume using the GraphPad Prism 10 and standards from the product literature indicated.

### 2.3. TPI Enzymatic Activity Assay

*Purified protein.* Enzymatic activity of recombinant TPI wildtype and R5G mutant were measured using an NADH-linked colorimetric TPI activity assay kit (Abcam, Cambridge, UK, product code: ab197001). Reactions were run in triplicate with protein concentrations of 1 ng, 5 ng, and 10 ng and according to the manufacturer’s instructions. Product formation was monitored at 450 nm with an Accuris SmartReader 96 microplate reader (Benchmark Scientific, Inc., Sayreville, NJ, USA).

*Cultured fibroblasts.* Protein activity was measured in lysates from FB909 cells using the same colorimetric assay kit as for purified protein above (Abcam, product code: ab197001). Cells were cultured as described in the Cell Culture section below, were plated in 10 cm Petri dishes, and allowed to attach overnight. A 50 µM compound **424** solution was prepared by adding 50 μL of 10 mM stock in DMSO to 9.95 mL 10% FBS DMEM. A vehicle control of 0.5% DMSO in 10% FBS DMEM was prepared accordingly. Medium was aspirated and cells treated for 48 h before harvesting cell pellets as described in the Cell Culture section. Cell pellets were resuspended in 10 mL of 10% FBS DMEM. 2 mL of the resuspension was used for BCA analysis while 8 mL was used for TPI activity assay. For the TPI activity assay, the supernatant was aspirated off, the pellet was resuspended in 2 mL PBS, and the resuspension was centrifuged at 3000× *g* for 5 min. The supernatant was aspirated off and the pellet was resuspended in ice-cold TPI assay buffer. The resuspension was transferred to 1.5 mL microcentrifuge tubes and kept on ice for 10 min. Afterwards, the microcentrifuge tubes were centrifuged at 10,000× *g* for 5 min. The lysate supernatant was collected into a new, clean microcentrifuge tube. Activity assay standards were prepared following the activity assay kit’s protocol. A standard curve with recombinant TPI (part of the assay kit) was prepared as per the manufacturer’s instructions and duplicates placed on the microplate. Additionally, a positive control was prepared by combining 40 μL TPI positive control compound with 60 μL TPI assay buffer. The positive control was added to the microplate in duplicates. 50 μL cell lysate samples were plated onto the microplate in duplicates. A master mix was created containing 44 μL protein assay buffer, 2 μL TPI enzyme mix, 2 μL TPI developer solution, and 2 μL TPI substrate per each well. The master mix was added to each well of the microplate. The microplate was kept on ice to prevent the reaction from beginning until being placed in the reader. The microplate was loaded into the reader (SpectraMax iD5 Multi-Mode Microplate Reader, Molecular Devices, San Jose, CA, USA) where the absorbance was measured at 450 nm every 3 min for 40 min at 37 °C. Absorbance values were normalized to lysate concentration and converted to NADH levels using the standard curve. The assay was performed independently twice by different technicians on different days with independently cultured and treated cells. Four technical replicates were performed each time. TPI activity was calculated per the manufacturer’s instructions using two time points (three minutes apart) in the linear portion of the reaction’s time course. Thus, the slope of the linear parts of the kinetic read curves was used to determine cellular TPI activity (in U/mg).

### 2.4. Thermal Shift Assay

Protein Thermal Shift Assay (PTS) was performed with a final protein concentration at 0.7 mg/mL for both TPI wildtype and R5G mutant in a reaction buffer of 20 mM HEPES pH 7.5, 150 mM NaCl, 10 mM Tris pH 8.8, 5% glycerol, and 1 mM β-ME. The hydrophobic dye added was 0.5X GloMelt. Four technical replications were run for each assay and the thermal gradient was applied from 30 to 95 °C using a Thermo-Fisher Quantstudio-3 Real-Time PCR machine (Thermo Fisher Scientific, Waltham, MA, USA). Fluorescence of GloMelt and ROX dye were measured, and the maximum derivative of fluorescence was used to define melting temperature (T_m_).

### 2.5. Cell Culture

Patient cells were obtained from a skin biopsy, were de-identified and cultured following University of Pittsburgh approved IRB 0404017. Cells were genotyped using standard sequence methods and named FB909, and aliquots were stored in liquid nitrogen for future studies. FB909 (R5G, frameshift mutation) cells were removed from liquid nitrogen and thawed in a 37 °C bead bath. The cells were diluted in 9 mL 20% FBS DMEM and centrifuged at 3000× *g* for 5 min. The supernatant was aspirated, and the cell pellet was resuspended in 5 mL 20% FBS DMEM, plated in a small flask, and placed into a 37 °C 5% CO_2_ incubator. After 24 h, the cell media were changed with 10% FBS DMEM. Cell media were changed every 3 days, and the cells were split depending on confluency. Upon confluency, the 10% FBS DMEM was aspirated off and PBS was washed over the flask. PBS was aspirated off the plate and trypsin was added. The plates with trypsin were incubated for 3 min to increase cell detachment. 10% FBS DMEM was added to the plate and the solution was pipetted to sterile 15 mL conical tubes. The solution was centrifuged at 3000× *g* for 5 min. The supernatant was aspirated off and the pellet was resuspended in 10% FBS DMEM. The solution was added to new plates with 10% FBS DMEM and the plates were placed in the incubator.

### 2.6. Cell Counting and Drug Treatment

Cells were first counted using a hemocytometer and plated with ~200,000 cells per plate. To count cells, the splitting procedure was followed up until the centrifugation step. After centrifugation, the cell pellet was resuspended with 10 mL 10% FBS DMEM. A measurement of 10 μL of the mixture was loaded onto the hemocytometer where cells were counted in 16-box grids and then were averaged to obtain a value of cells per microliter in solution. Since 200,000 cells were required for each plate, 200,000 was divided by the number of cells per microliter to determine how many microliters were needed to obtain 200,000 cells per plate. An additional 10% FBS DMEM was added to the plate to reach a final volume of 10 mL. The plates were placed in the incubator.

After 24 h, the media were aspirated from the plates. New media with compound **201**, **424**, **846**, or DMSO was added to each plate. A measurement of 50 μL of 10 mM compound **424** or **201**, 10 μL of 10 mM compound **846**, or DMSO was combined with 9.95 mL 10% FBS DMEM. The solutions were then added to cell plates for 48 h. The cell plates with drug treatments were placed in the incubator.

### 2.7. Immunofluorescence in TPI-Df Patient Cells

Patient fibroblasts were plated in collagen-coated Revvity Phenoplates, allowed to attach overnight, and treated with test agents in ten point, two-fold compound gradients. Forty-eight hours after treatment, cells were fixed with 4% formaldehyde for 15 min at room temperature. Cells were permeabilized with 15 µL blocking buffer (0.3% Triton X-100 and 1% BSA in PBS, 0.45 µm filtered) containing 10 µg/mL Hoechst 33,342 for one hour at room temperature, followed by the addition of 15 µL of primary antibody (1:1000 Rabbit poly PA5-21583 WH3264660A (Invitrogen, Carlsbad, CA, USA)) overnight at 4 °C. Plates were washed three times with PBS and incubated with 1:500 Cy3-conjugated AffiniPure Donkey Anti-Rabbit IgG (H + L) secondary antibody (Jackson Immunoresearch, West Grove, PA, USA, 711-165-152) in blocking buffer for 45 min at room temperature. Plates were washed with PBS, sealed, and stored at 4 °C until analysis. Plates were imaged on an OPERA Phenix Plus high content reader (Revvity, Inc., Waltham, MA, USA) with a 20× air objective, laser lines, and emission filters for Hoechst (ex405/em435–480 nm) and Cy3 (ex561/em570–630 nm). Images were analyzed and archived in Harmony 5.1.

### 2.8. Western Blot

Cells were treated with compound **201** (50 µM), **424** (50 µM), **846** (10 µM), or DMSO as described in the TPI Activity section above. Cells were lysed and analyzed for protein content by the BCA method as described above, except that a RIPA lysis buffer with protease inhibitors was used. The trypsin 10% FBS DMEM supernatant was aspirated off and the pellet was resuspended in PBS. The resuspension was centrifuged at 3000× *g* for 5 min. The supernatant was aspirated off and the cell pellet was resuspended in 120 μL of RIPA buffer + protease inhibitor solution. The resuspension was transferred to 1.5 mL microcentrifuge tubes and kept at −80 °C.

SDS PAGE samples were prepared by combining 20 μL of 2× Laemmli Sample Buffer with 20 μL of lysate from cell harvest. Each sample was heated for 5 min then centrifuged briefly to remove condensation. Samples were then loaded into a precast gel, and volume was calculated from the BCA analysis. A measurement of 5 μL of the stained protein molecular weight standard was loaded into the first well of the gel. The gels were run at 200 V for 50 min. To facilitate the protein transfer, a sandwich method of two wet blot papers, a PVDF membrane, the gel, and an additional wet blot paper was used. All components were wet with transfer buffer, stacked, then rolled out to avoid bubbles. The transfer was run at 12 V for 3 h.

After transfer, the blot was blocked for one hour at room temperature in 10 mL Odyssey Blocking Buffer with the protein side face-up in the buffer. After blocking completion, the blot was incubated with 3 mL of primary antibody solution in blocking buffer overnight at 4 degrees Celsius. The primary antibody solution consisted of 0.6 μL TPI antibody (Fisher, Waltham, MA, USA, PIPA521583) and 3 μL beta tubulin antibody (DSHB, Iowa City, IA, USA, E7-c) in 3 mL blocking buffer.

The following day, each blot was washed 6 times for 5 min each in PBS-T. After washing, the blot was incubated in secondary antibody solution at room temperature in the dark for 2 h. The secondary antibody solution consisted of 10 μL Tween 20, 0.5 μL goat anti-mouse (Fisher, A21121), 0.5 μL donkey anti-rabbit (Fisher, NC9523609), and 10 mL blocking buffer. After incubation, the gels were washed in PBS-T for an additional 6 times for 5 min each. After washing, the blots were imaged and analyzed using a LICOR machine.

### 2.9. Data Analysis

Statistical tests were performed using GraphPad Prism 10 (version 10.6.1). For specific in vitro activity experiments, unpaired *t*-tests were used to determine significance. In vitro pecific activity measurements were performed with three technical replicates using three different quantities of TPI for a total of nine measurements. Differential scanning fluorometry results for TPI thermal stability are the result of four technical replicates and a two-sample one-tailed unpaired *t*-test was used to determine significance. Western blot and cellular activity data were analyzed using a two-tailed Mann–Whitney test for significance. All Western blot data consist of eight independent replicates. For all analyses, the error depicted is standard deviation.

## 3. Results

### 3.1. Clinical Presentation

The patient is a 15-year-old female with a history of dystonia and developmental delays, which include cognitive and speech delays, as well as unsteadiness and motor regression. She was born at 37 weeks of gestation with forceps and a broken collarbone. She could not sit independently at 9 months of age and was first evaluated at 13 months due to progressive loss of motor function and painful, generalized dystonia following a high fever. Her family medical history proved to have no underlying causes as both parents and both sisters were in good health. Initial biological work-up showed no signs of abnormality. There were no hematological abnormalities, liver function tests were normal, and renal functions and creatine kinase levels were normal. Plasma and urine analysis was normal and showed no signs of changes in composition. Cerebrospinal fluid (CSF) metabolic screening, which measured amino acid and organic chromatography, lactate, and CSF neurotransmitter levels were all normal. Furthermore, no CSF oligoclonal band was found and auto-neuronal antibodies were negative. Initial MRI, EEG motor, and sensory velocities were normal. Initial genetic analysis showed that both single-gene and panel (dystonia) sequencing were not conclusive for the following genes: *ATP1A3*, *PRKRA*, *CACNA1a*, *DYTPRI*, *DYT1*, and *DAT1*. As she grew older, the patient’s motor functions did progress, but were significantly delayed compared to healthy individuals her age.

At age 10, control brain MRI revealed bilateral abnormal signals in the basal ganglia, with the posterior aspect of the putamen and the claustrum appearing slightly hyperintense. Atrophy of the putamen, mainly on the posterior side, was noted. There was no abnormal contrast enhancement. The spectroscopy study did not show a lactate peak. Compared to previous MRIs, a slight increase in atrophy was observed.

Molecular analysis via whole-exome sequencing was conducted and revealed compound heterozygous variants from the two asymptomatic heterozygous parents, at the *TPI1* gene. The variant of paternal origin (c.452_453del, p. (Ile151Serfs*3)) has been considered as likely pathogenic (class IV), while that of maternal origin (c.13A>G, p. (Arg5Gly)) of undetermined significance (class III) was potentially considered as pathogenic. TPI enzyme activity was decreased in the child’s erythrocytes (182 Ug Hb between 13 and 30% of normal values (nl 633–1348)). Both parents had intermediate values (mother 798 U/g Hb; father: 756 U/g Hb).

At 12 years of age, the child experienced several migraine episodes with loss of sensation in the left lower and upper limbs, left facial paresis with anarthria but without loss of consciousness, followed by right frontal headaches with complete recovery after several minutes. Her treatment was progressively adapted as follows: riboflavin, magnesium ubidecarenone, Resveratrol, propranolol, and drospirenone.

At age 15, she lost the ability to walk independently. She maintained an asymmetrical dystonic gait, more pronounced distally and proximally on the left and at the facial level. Grip and grip strength were difficult on the right due to a flexed hand. She had numerous jerks, ranging from choreic to myoclonic. Osteo-tendinous reflexes were increased, but without Babinski signs. Her speech was only partly comprehensible because of the dysarthric component. Her weight was 64 kg (<p 90) for a height of 159.0 cm (<p 50) and head circumference of 56.9 cm (2 SD). Her BMI was 25.1 kg/m^2^ (P92).

### 3.2. Mutational Analyses

**Figure 1:**
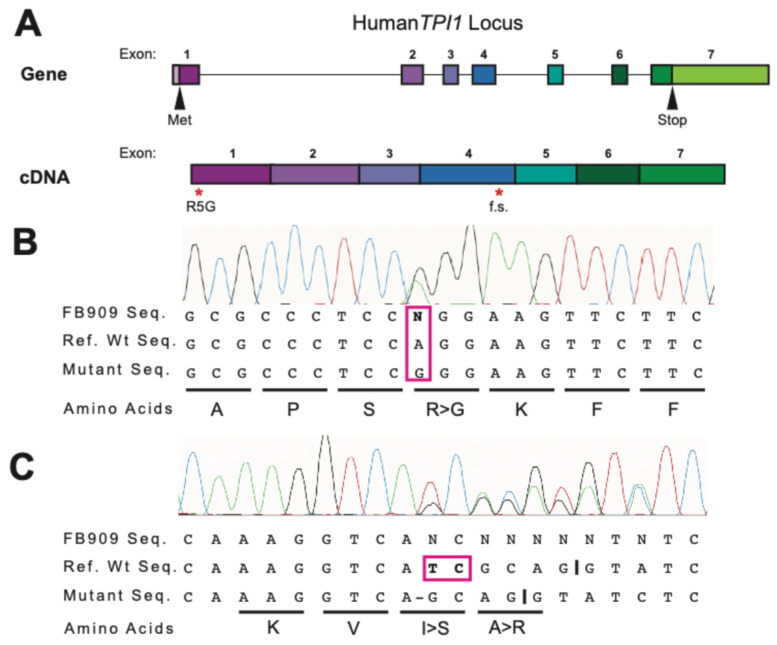
Sequence Analysis of *TPI^R5G/f.s^* alleles. (**A**) Human *TPI1* genomic locus map indicating the location of the *R5G* and *frameshift* (*f.s.*) mutations. (**B**) Sequence chromatograph from patient FB909 cells indicating the heterozygous *R5G* mutation (highlighted by magenta box). (**C**) Sequence chromatograph from patient FB909 cells indicating the *f.s.* mutation. The dash indicates the location of the 2-nucleotide deletion that results in a frameshift. Vertical lines represent the exon–intron junction. The *f.s*. mutation results in I151S, A152R, and D153W followed by a premature termination codon at position 154.

The patient was found to have a novel *TPI* mutation which differs from previous *TPI1* variants such as *TPI^E105D^*, *TPI^R190Q^*, and *TPI^Q181P^*. The patient was determined to be heterozygous for two *TPI1* mutations (p.R5G and f.s.). The novel *TPI^R5G and^ TPI^f.s.^* variants identified clinically by whole-exome sequencing were confirmed from patient-derived fibroblasts. The *TPI1* locus was PCR-amplified from FB909 cells and Sanger sequencing was performed. Sequencing results showed both a point mutation at the fifth amino acid position and a frameshift mutation at codon 151 in which 2 nucleic acids were deleted (Figure 1), confirming the clinical result and the integrity of the FB909 cells.

### 3.3. TPI^R5G^ Protein Biochemistry

**Figure 2:**
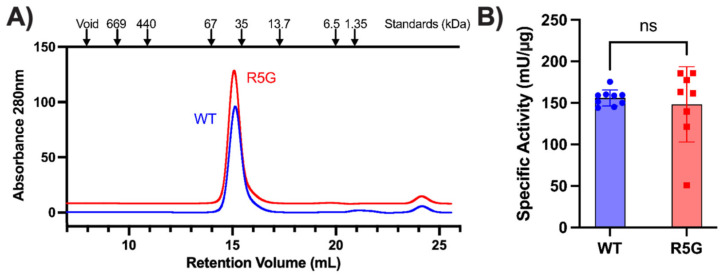
Biophysical Characterization of TPI^R5G^. (**A**) Analytical size-exclusion chromatography of WT and R5G TPI variants. Standards are indicated on the top axis. (**B**) Specific TPI activity for WT and R5G mutant TPI. Data points are the measurements for specific activity from assays containing 1, 5, or 10 ng of the indicated TPI protein. Unpaired *t*-test was used to determine significance, *n* = 8, error bars indicate Std. Dev. ns, not significant.

**Figure 3:**
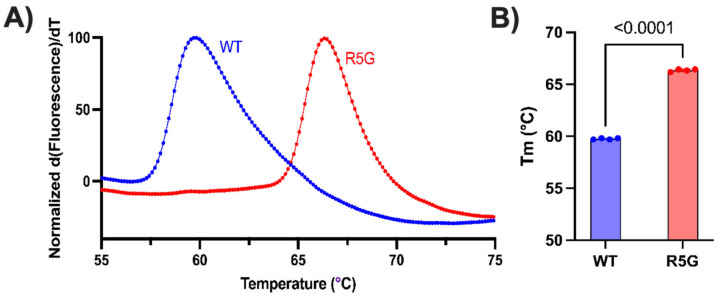
Thermal stability of TPI R5G protein. (**A**) Thermal denaturation from Differential scanning fluorometry of WT (blue) and R5G (red) variants of TPI. Curves representing the derivative of fluorescence were used to obtain melting temperatures (TMs). The curves shown represent the average of four technical replicates. (**B**) The individual TMs for each protein are plotted in B with the bars representing the average. Two-sample one-tailed unpaired *t*-test was used to determine significance, *p* < 0.0001, error is Std. Dev.

The novel *TPI^R5G^* variant was identified in a compound heterozygous patient with a frameshift allele. The delta 2 NT deletion at codon 151 is in exon 4 and, since this is not the last exon, nonsense-mediated decay should destabilize the mutant mRNAs, resulting in their degradation. Additionally, the mutation is predicted to put the last ~100 codons of this protein out of frame for translation and, if the mRNAs were to be translated, they would certainly lack isomerase activity. Thus, the delta 2 NT *f.s.* mutation is predicted to be a *TPI1* null allele. However, the novel *TPI^R5G^* variant is of uncertain consequence as it is not a known polymorphism, or a mutation described in the literature or in available databases. To better understand the effect of the missense mutation resulting in TPI^R5G^, we used a bacterial expression system to purify TPI^R5G^ and TPI^WT^ proteins for biochemical interrogation. The purified proteins were examined by size-exclusion chromatography, and both were found to exist as dimers (Figure 2A). The R5 amino acid is positioned adjacent to the helix which contains Q181P and R190Q, both of which are pathogenic amino acid substitutions which result in decreased TPI activity and TPI Df in compound heterozygous patients with the common *TPI1^E105D^* allele [8,9]. Therefore, we performed TPI activity assays with both WT and R5G mutant proteins to investigate whether the R5G amino acid substitution affects catalytic activity of the protein. Interestingly, while there was more experimental variability, there was not a statistically significant difference in specific isomerase activity in TPI^R5G^ when compared to wildtype protein (Figure 2B). Lastly, we examined the thermal stability of the dimer using Differential scanning fluorometry (also known as a protein thermal shift assay). This assay subjects the protein to a thermal gradient in the presence of a hydrophobic dye, detecting denaturation of the protein as a rapid increase in fluorescence when the hydrophobic core of the protein becomes exposed. Using this assay, we observed a significant increase in the stability of the TPI^R5G^ mutant dimer over wildtype protein (Figure 3). These data suggest that the mutant protein is more resistant to opening of the hydrophobic core, which has also been observed in other pathogenic TPI Df alleles, including *TPI^I170V^*, *TPI^Q181P^*, and *TPI^R190Q^* [8,9].

### 3.4. Mutant TPI Protein Levels

**Figure 4:**
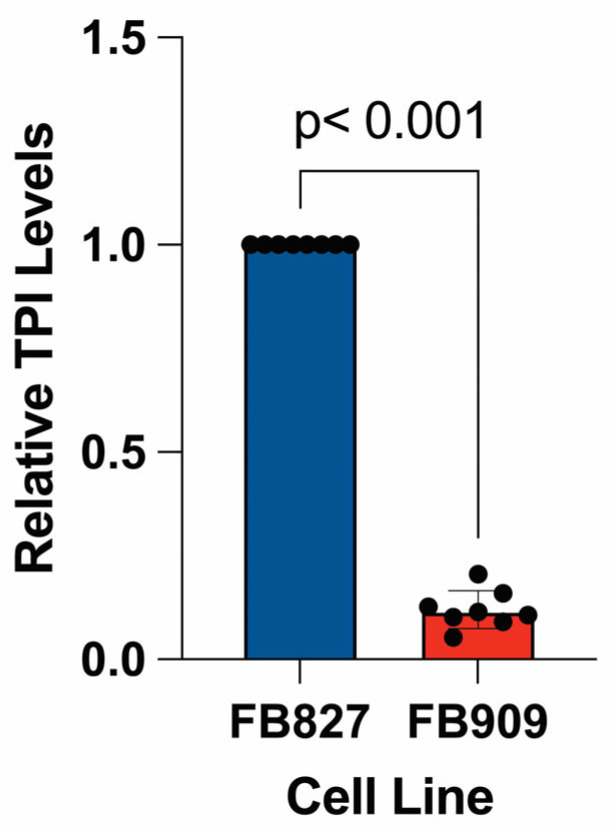
Western blot of TPI from whole protein extracts. FB909 patient-derived fibroblast cells (R5G/f.s) exhibit a dramatic ~90% reduction in TPI protein compared to control fibroblast cells FB827. Two-tailed Mann–Whitney test was used to determine significance, *n* = 8 for each cell line, error is Std. Dev.

To determine the basal level of expression in *TPI^R5G/f.s.^* patient fibroblasts (FB909), we measured TPI levels using Western blot. We discovered a marked reduction in TPI protein levels in patient-derived fibroblasts compared to wildtype cells (Figure 4). The expectation is that the frameshift mutation is responsible for ~50% reduction in steady-state protein levels. Thus, since there is an ~90% decrease in TPI protein relative to control fibroblasts, these data demonstrate that the novel TPI^R5G^ mutant protein has pathogenic consequences that significantly decrease either its expression or stability, lowering steady-state protein levels. Notably, other pathogenic alleles of *TPI1* that lead to TPI Df have been described where the mutant protein retains significant biochemical function and thermal stability but exhibits a decrease in steady-state protein levels due to accelerated turnover [1,8,9].

### 3.5. Lead TPI Df Compounds 201, 424, and 846

**Scheme 1:**
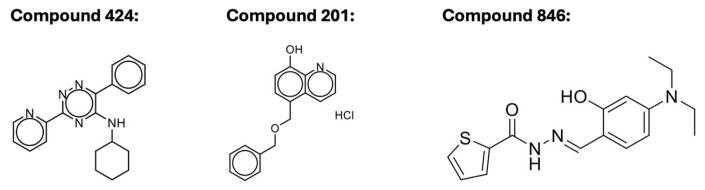
Structures of compounds proposed as TPI Df lead therapeutics.

Previously, a stable human cellular model of TPI Df expressing the common TPI^E105D^ protein fused to eGFP was developed [13]. Recently, this model was further developed into an assay to screen ~225 k compounds to identify novel lead compounds as TPI Df treatments [14]. Three hit compounds identified in the screen were shown to be effective in patient cells homozygous for the common *TPI^E105D^* mutation [14]. Here we report the study of those three compounds in FB909 cells with a novel disease-causing genotype (Scheme 1). Compound **846** (MolPort-000-442-846) is a carbohydrazide. Compound **424** (Molport 002-877-424) is a 1,2,4-triazine. Compound 201 (Molport 047-151-201) is an 8-hydroxyquinoline. The structures of these compounds are shown in Scheme 1.

### 3.6. Compounds 201, 424, and 846 Increase TPI Levels in TPI^R5G^ Cells

**Figure 5:**
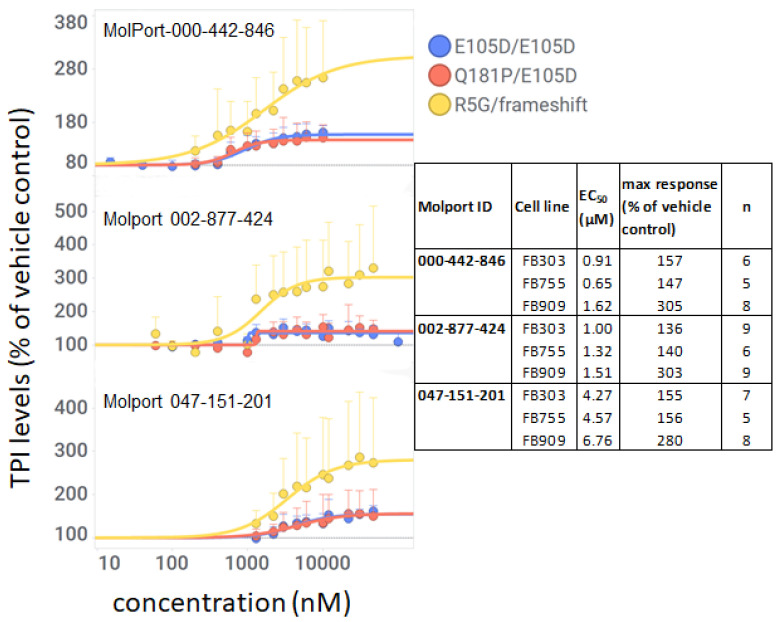
Comparative activity of compounds **846**, **424**, and **201** in fibroblasts from different genotype donors. TPI Df patient fibroblasts FB303 (E105D/E105D, “common mutation”, blue), FB755 (Q181P/E105D, red), FB909 (R5G/f.s., yellow) were treated for 48 h with ten-point, two-fold gradients of compounds and analyzed for cytosolic TPI levels by immunofluorescence on an OPERA Phenix Plus HCS reader. Data points represent the average TPI intensity, normalized to vehicle controls, of the indicated number of independent biological repeats ± SD. Aggregated curves were fitted to a four-parameter logistic equation in Spotfire (Ver 14.0.2, Revvity, Inc.). Inset table summarizes EC_50_ values and maximum increases in TPI levels. All compounds had similar EC_50_s in all three cell types, but their magnitude of response varied, with FB909 showing the largest maximum % increase.

**Figure 6:**
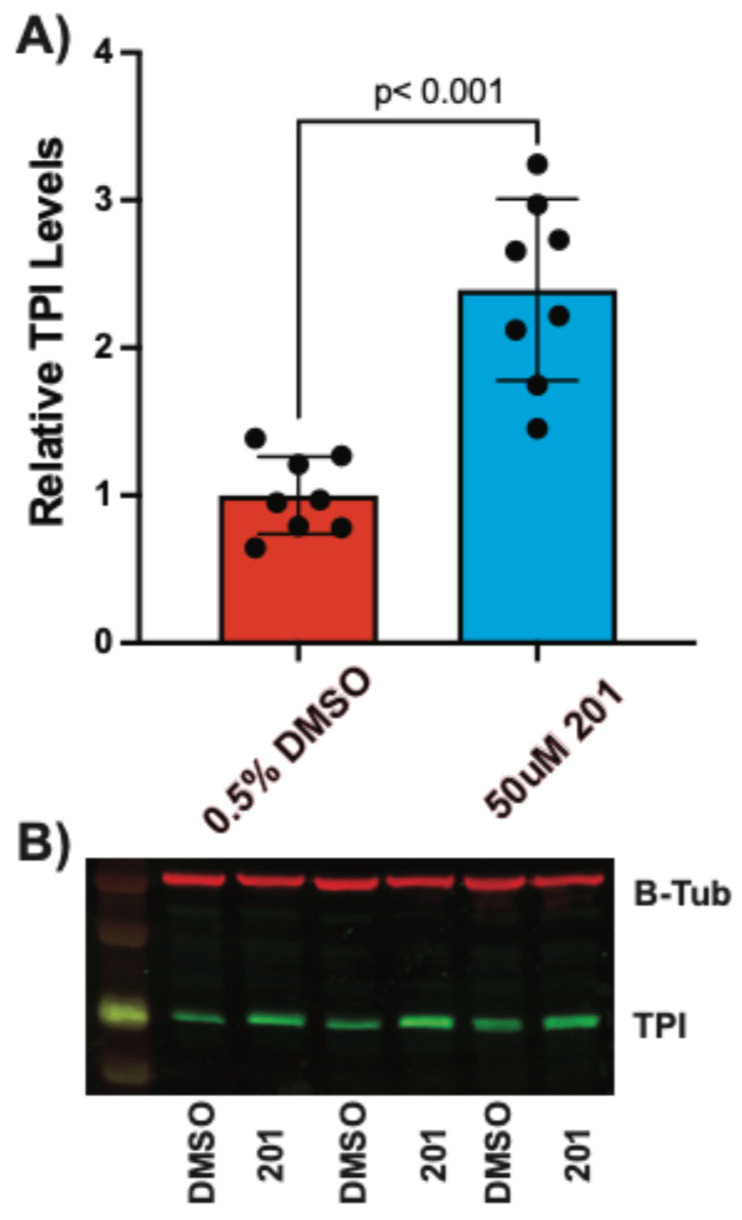
Compound **201** increases TPI protein levels in human fibroblasts. (**A**) Western blot analysis demonstrates significant ~240% average increase with 50 μM **201** treatment compared to DMSO-treated controls. (**B**) Representative blot used in 50 μM **201** treatment analysis. Two-tailed Mann–Whitney test was used to determine significance. *p*-values are shown, *n* = 8 for each group, error is Std. Dev.

**Figure 7:**
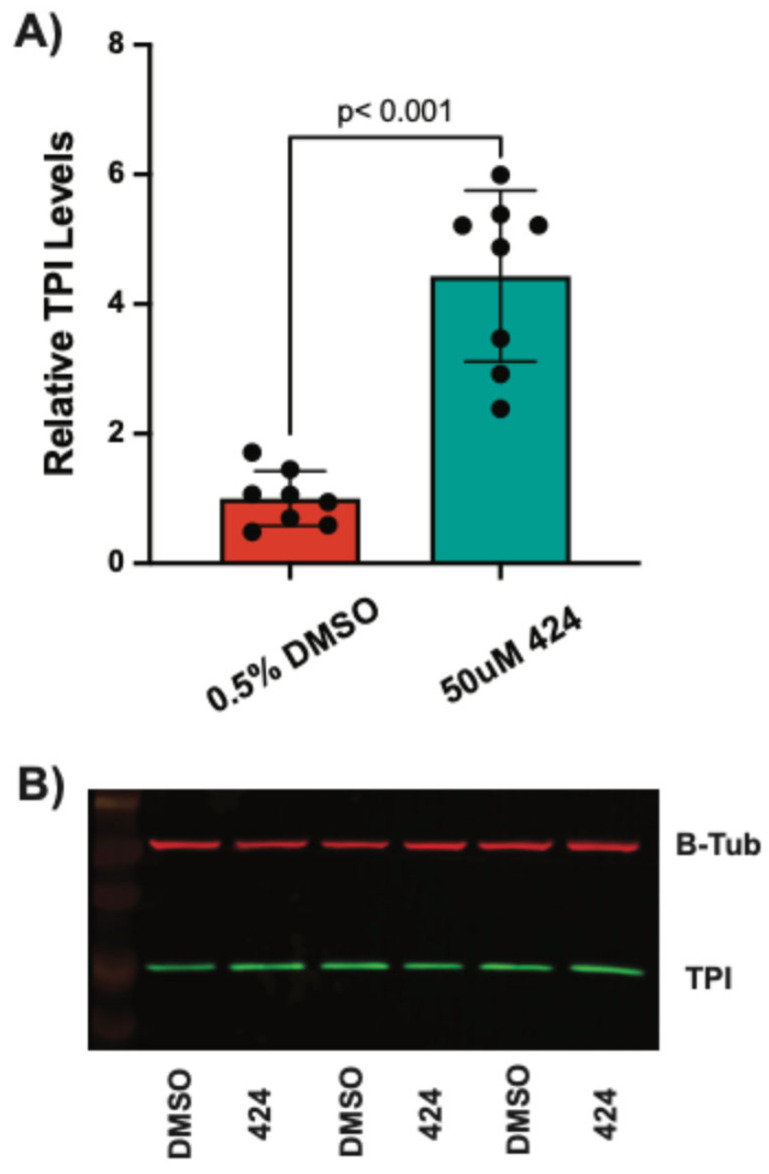
Compound **424** increases TPI protein levels in human fibroblasts. (**A**) Western blot analysis demonstrates significant ~410% increase with 50 μM **424** treatment compared to DMSO-treated controls. (**B**) Representative blot used in 50 μM **424** treatment analysis. Mann–Whitney test was used to determine significance. *p*-values are shown, *n* = 8 for each group, error is Std. Dev.

**Figure 8:**
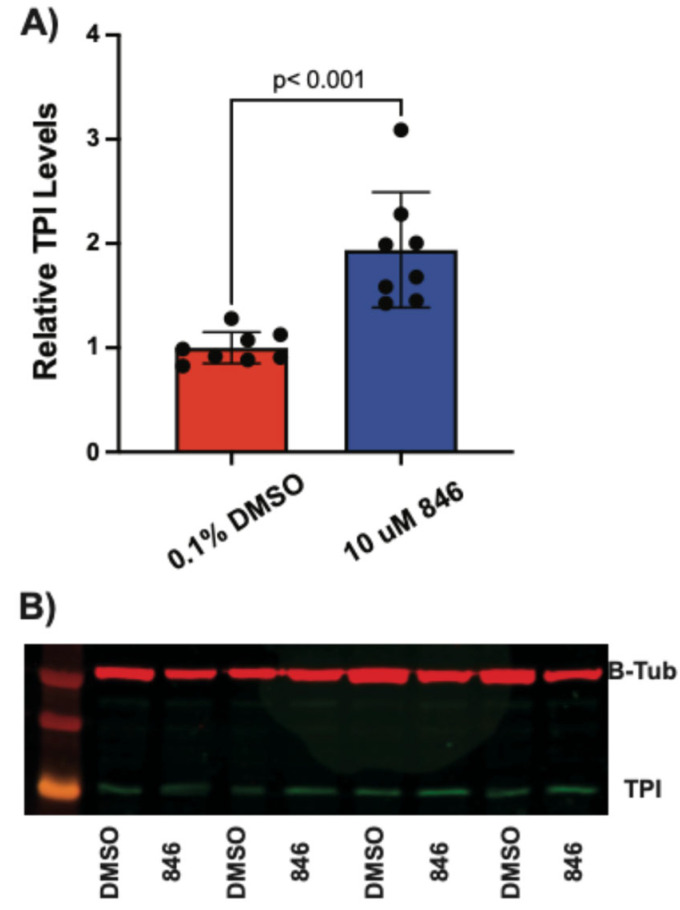
Compound **846** increases TPI protein levels in human fibroblasts. (**A**) Western blot analysis demonstrates significant ~190% average increase with 10 μM **846** treatment compared to DMSO-treated controls. (**B**) Representative blot used in 10 μM **846** treatment analysis. Two-tailed Mann–Whitney test was used to determine significance. *p*-values are shown, *n* = 8 for each group, error is Std. Dev.

To investigate whether these compounds increased levels of mutant TPI in FB909 cells and, if so, whether they showed any selectivity for specific TPI alleles, we performed immuno-histochemistry (IHC) in TPI Df patient cells with different mutant TPI genotypes using a large range of concentrations. All three compounds increased TPI levels in FB909 cells by IHC with EC_50_ values of 1.62, 1.51, and 6.76 µM for **201**, **424**, and **846**, respectively (Figure 5 and table within). EC_50_s were similar in all three cell types (Figure 5), however their magnitude of response varied. Whereas fibroblasts with either the common mutation or the *TPI1^Q181P/E105D^* mutation responded with less than 2-fold maximum increases, the FB909 cells showed a much larger maximum response (~3-fold). These results were surprising since FB909 cells were predicted to contain a null allele and only express protein from one chromosome. We then performed Western blots as an orthogonal assay to verify the effect of these compounds. All three compounds produced significant increases in TPI levels in FB909 cells compared to vehicle-treated controls (Figure 6, Figure 7 and Figure 8). Compound **424** produced an ~4-fold increase (Figure 7), whereas compounds **201** and **846** produced ~ 2-fold increases (Figure 6 and Figure 8), confirming the results from the IHC assays.

### 3.7. Compound 424 Increases TPI Activity in FB909 Cells

**Figure 9:**
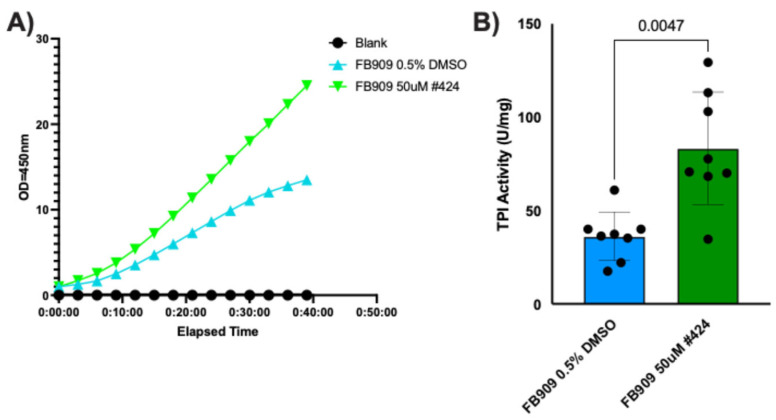
TPI activity in FB909 cell lysates treated with compound **424**. (**A**) Example kinetic graph showing changes in absorbance in samples treated with 50 μM **424** compared to control. Time was standardized to T = 0. (**B**) TPI protein analysis shows a significant increase in protein lysate activity (U/mg) when treated with 50 μM compound **424** compared to control. Two-tailed Mann–Whitney test was used to determine significance. *p*-values are shown, *n* = 8 for each group.

Purified TPI^R5G^ protein has specific activity that is not different than wildtype TPI protein (Figure 1B), suggesting that increases in TPI protein levels observed could significantly increase cellular TPI activity. As compound **424** produced the largest increase in steady-state TPI protein levels, we measured whether this compound significantly increased TPI activity in patient cells. Cellular TPI activity was analyzed using a commercially available NADH-linked colorimetric activity assay (Abcam). FB909 patient-derived fibroblasts were treated with compound **424** (50 μM) for 48 h, harvested, and lysed. Cell lysates were split for bicinchoninic acid assays (BCA) to determine total protein and for TPI activity assays. Compared to the DMSO control, treatment with **424** substantially increased absorbance over time (Figure 9A). For a quantitative assessment of TPI activity, absorbance data were normalized to total cell lysate and TPI activity (in U/mg) was calculated from kinetic curves as described in the Section 2. Total cellular isomerase activity increased ~2-fold with 50 μM **424** treatment (Figure 9B).

## 4. Discussion

TPI Df is an ultra-rare pediatric disease characterized by anemia and severe neuromuscular dysfunction that is both debilitating and contributes to an early death, typically prior to adolescence. The “common” *TPI^E105D^* mutation is found in almost all patients, most often homozygous but not infrequently with other alleles that accelerate/worsen the disease or lead to less severe symptoms and a much longer lifespan for some patients [8,9]. Here we describe a patient with two previously unreported alleles, *TPI^R5G/f.s.^*, with a unique form of TPI Df that lacks anemia and recurrent episodes of infection, is less severe and more slow-progressing than the “common” *TPI^E105D/E105D^*presentation, yet still results in severe neurologic symptoms including dystonia. To better understand TPI Df and disease pathogenesis in this patient, biochemical studies were performed on the novel TPI^R5G^ protein and patient cells.

The novel *TPI1^R5G^* mutation was identified in a patient with a frameshift allele at codon 151 that is a null allele and does not encode a functional isomerase. Consistent with TPI being an essential enzyme in all organisms from *E. coli* to human, all known TPI Df patients have had a missense allele (or two) that encodes a protein which retains significant isomerase activity. Most commonly, the disease-causing missense allele encodes a protein with full or nearly full isomerase activity; however, the protein is less abundant in the cell, likely due to reduced stability and accelerated turnover of the mutant protein, as has been shown in animal models of the disease [10,11,12]. Interestingly, the TPI^R5G^ protein not only results in severe deficits clinically but also acts like other known *TPI1* mutations on a cellular and molecular level. Both wildtype and mutant TPI^R5G^ proteins exist as dimers. Purified TPI^R5G^ protein exhibited no difference in specific isomerase activity compared to purified wildtype TPI protein. However, there was a significant increase in the stability of the mutant TPI^R5G^ dimer protein than the wildtype protein. We do not know the mechanism by which steady-state protein levels are decreased. The increased thermal stability of the R5G dimer may indicate that folding is altered or may lead to an acceleration in degradation. Whatever the case, these data suggest that the R5G mutant protein is similar to other identified pathogenic TPI mutant proteins. Specifically, TPI Df-associated missense alleles including *E105D*, *Q181P*, and *R190Q* all exhibit reduced protein levels, like *R5G*; however, E105D retains high levels of activity and Q181P has very low activity (~10% of WT) [1,15,16,17]. Since all these pathogenic mutations decrease protein levels and preserve at least some catalytic activity, a therapy that stabilizes protein levels would likely work broadly for most patients.

Currently, TPI Df does not have a treatment beyond dietary restrictions, nutrient supplementation, and supportive care. TPI Df is an ultra-rare disease and is unlikely to become the focus of commercial drug development. To advance therapies for TPI Df, human cellular models of the disease have been developed that allow the screening of test compounds [13]. Recently, one such screen was developed into a High Throughput Screen that screened > 225k compounds and resulted in three novel series of compounds that significantly increased TPI levels without increasing ROS or exhibiting overt signs of apoptosis in a HeLa cell model of mutant TPI [14]. Three of the compounds were validated in TPI Df patient cells that were homozygous for the common mutation and include a hydroxyquinoline (compound **201**), a triazine (compound **424**), and a hydrazone (compound **846**) [14]. The mechanism of action for these three drugs has not been rigorously established but there is evidence that they could be acting by more than one mechanism, based upon their metal chelation properties and ability to inhibit the proteasome [14]. Therefore, these are promising lead compounds deserving of further development for TPI Df, but which have previously only been tested on cells expressing the common *TPI^E105D^* protein. Therefore, we examined the effect of all three hit compounds on FB909 patient cells with the *TPI^R5G/f.s.^*genotype. All three compounds increased TPI levels measured in FB909 cells, with EC_50_ values in the low micromolar range (Figure 5). Interestingly, all three of these compounds produced similar ~ 50% increases in TPI protein levels in FB303 cells homozygous for the common mutation and FB755 cells with a Q181P/E105D mutation (Figure 5), whereas, in FB909 cells the percent increase in TPI protein was ~ 300%. Western blots revealed slight differences in TPI levels among the compounds, with compound **424** showing the largest effect (~4-fold increase over vehicle control (Figure 6, Figure 7 and Figure 8)). The TPI^R5G^-purified protein has essentially wildtype activity in vitro (Figure 3), suggesting that these increases in protein levels would result in more isomerase activity in cells. We examined activity in FB909 cells treated with **424** and observed a striking increase in TPI activity in patient fibroblasts (Figure 9).

## 5. Conclusions

The data reported here demonstrate three promising lead compounds that increase TPI levels and that increases in TPI levels result in increased isomerase activity. Importantly, these compounds show efficacy in the context of several TPI Df patient cells, including the one reported here with the novel *TPI^R5G/f.s^*^.^ genotype. Future studies will involve testing a series of analogs of these lead compounds, exploring their mechanisms of action, and in vivo efficacy testing of these compounds using an established TPI Df murine model [16,18]. The compounds produce very significant increases in TPI protein and activity in patient cells, which is encouraging. One limitation of these is that all the data reported here are in vitro, and in vivo efficacy has not been established. A viable therapeutic compound for TPI Df will need to have good penetration throughout the neuromuscular system and function broadly in patient tissues. Thus, drug distribution studies to specifically measure the penetration of all muscle types, the pharmacokinetic properties of the drugs, and the ability of the compounds to cross the blood–brain barrier will be important to prioritize in future in vivo efficacy studies. Importantly, if these lead compounds function in vivo, they then treat the underlying cause of the disease, insufficient TPI protein, and could potentially significantly correct TPI Df rather than simply mitigate symptoms of the disease.

## Data Availability

The raw data supporting the conclusions of this article will be made available by the authors on request.

## Abbreviations

The following abbreviations are used in this manuscript:

TPI	Triosephosphate Isomerase
MGO	Methylglyoxal
HTS	High Throughput Screen
